# The effects of virgin coconut oil (VCO) as supplementation on quality of life (QOL) among breast cancer patients

**DOI:** 10.1186/1476-511X-13-139

**Published:** 2014-08-27

**Authors:** Kim Sooi Law, Nizuwan Azman, Eshaifol Azam Omar, Muhammad Yusri Musa, Narazah Mohd Yusoff, Siti Amrah Sulaiman, Nik Hazlina Nik Hussain

**Affiliations:** Institut Perubatan dan Pergigian Termaju, Universiti Sains Malaysia, Bertam, 13200 Kepala Batas, Pulau Pinang Malaysia; School of Medical Science, Health Campus, Universiti Sains Malaysia, Kelantan, Malaysia

**Keywords:** Virgin coconut oil (VCO), Quality of life (QOL), Breast cancer patient, Chemotherapy

## Abstract

**Background:**

Breast cancer is the most common cancer amongst Malaysian women. Both the disease and its treatment can disrupt the lives of the woman and adversely affect all aspects of life and thus can alter a woman’s quality of life. The aim of this study was to examine the effect of virgin coconut oil (VCO) on the quality of life (QOL) of patients diagnosed with breast cancer.

**Methods:**

This was a prospective study of breast cancer patients admitted into the Oncology Unit of Hospital Universiti Sains Malaysia, Kubang Kerian, Kelantan, Malaysia. The sample consisted of 60 patients with stage III and IV breast cancer allocated to either an intervention group (n = 30) or a control group (n = 30) using a simple random table. QOL was evaluated from the first cycle of chemotherapy to the sixth cycle, and data were collected using a validated *Bahasa Malaysia* version of the European Organization for Research and Treatment of Cancer Quality of Life Questionnaire Breast Cancer Module (EORTC QLQ-C30) and its breast-specific module (QLQ-BR 23).

**Results:**

The mean age of breast cancer patients was 50.2 (SD = 13.5) years. There were significant mean score differences for functioning and global QOL between groups (α < 0.01). The intervention group also had better scores for symptoms including fatigue, dyspnea, sleep difficulties, and loss of appetite compared to the control group. Although there are deteriorations for sexual enjoyment, the intervention group exhibited improvement in breast functioning and symptom scores for body image, sexual function, future perspective, breast symptoms, and systemic therapy side effects.

**Conclusion:**

VCO consumption during chemotherapy helped improve the functional status and global QOL of breast cancer patients. In addition, it reduced the symptoms related to side effects of chemotherapy.

## Background

In Malaysia, breast cancer is the most common cancer that affects women and rated as the main cause of mortality, among other cancers [1]. According to the third report of the National Cancer Registry (2003–2005), the incidence of breast cancer among women in Malaysia is 31.3%. The high mortality rate of breast cancer also contributed to the 15.2% of medically certified death due to cancer [2].

Chemotherapy and radiotherapy are commonly used after primary treatment of breast cancer to inhibit metastasis and enhance long-term survival rates [3–6]. However chemotherapy often is associated with many negative side effects such as nausea and vomiting, hair loss, fatigue, pain, anxiety, depression and others [7–9]. Most of the side effects were perceived by the patients as distressing and many were anxious during the treatment. Both the disease and its treatment can disrupt the lives of the woman and adversely affect all aspects of life and thus can alter a woman’s quality of life (QOL) [7, 10–15]. Akin *et al*., [16] demonstrated that the adverse effects of different cancer or treatment-related symptoms and types of treatment have some correlations with QOL. Furthermore, decreased health-related QOL may affect daily life in patients with breast cancer [17].

Virgin coconut oil (VCO) is a traditional product that has a long history of ethnopharmacological use. VCO is extracted from fresh coconut flesh at low temperature and without the use of chemicals. Studies have shown that coconut oil inhibits the induction of carcinogenic agents in the colon and in mammary tumours in test animals [18–20]. In chemically induced cancers of the colon and breast, coconut oil was shown to be more protective than unsaturated oil. Coconut oil which is rich in medium chain fatty acids, and ideal for immune suppressed individuals. For this reason, it is being studied as a treatment for HIV/AIDS patients whose immune systems are severely compromised [21]. In a clinical study conducted by Soerjodibroto [22] of the effects of VCO on immune responses among HIV positive patients, he concluded that the macronutrient intake, mostly in terms of energy, fats and protein were significantly improved among the VCO supplemented group compared to the control group. In addition, the weight and nutritional status of the subjects, especially among the VCO-supplemented group, were maintained well throughout the study.

To date, studies on the use of VCO as a dietary supplement in the treatment of cancer patients to improve their QOL are limited. However, this information could help healthcare professionals care for cancer patients and help them maintain a good QOL during treatment. Thus, the purpose of this study was to empirically evaluate the effect of VCO supplementation on the QOL of breast cancer patients undergoing chemotherapy in Kelantan, Malaysia.

## Methods

### Study design

This was a prospective study of health-related QOL of breast cancer patients. The study was conducted in the Hospital Universiti Sains Malaysia (HUSM) located in Kelantan, Malaysia. HUSM is a tertiary and teaching hospital in Malaysia. The sixty participants included in this study were women ≥ 18 years old who were diagnosed with stage III or IV breast cancer and who underwent six cycles of first-line chemotherapy.

Pre-assessment using the study instrument was conducted among patients who met the study inclusion criteria during their first visit for their first cycle of chemotherapy. During their second cycle of chemotherapy, patients were randomly assigned to either the intervention or control group using a simple random sampling. Patients in the intervention group (n = 30) were given VCO (10 ml twice daily) as a supplement after 1 week each chemotherapy cycle from the third until the sixth cycle, whereas patients in the control group did not receive any supplement.

Ethical approval for the study was obtained from the Human Research Ethics Committee, USM and the Director of HUSM. The patients were informed about the study and the informed consent was obtained from the patients.

### Outcome measures

QOL was measured using the translated *Bahasa Malaysia* version of the European Organization for Research and Treatment of Cancer Quality of Life Questionnaire–Core 30 (EORTC QLQ-C30) and the Breast Cancer Supplementary Measure (QLQ-BR23) questionnaire. The psychometric properties of the translated version for both questionnaires have been well documented [23, 24].

The EORTC QLQ-C30 is a well-known instrument for measuring QOL in cancer patients. It contains 30 items that measure the following three main domains: global health status, functional status, and cancer-related symptom status. The QLQ-BR23 is a breast cancer-specific set of 23 questions for assessing functional status and symptoms related to breast cancer. The EORTC raw score of a participant’s responses is transformed according to the EORTC scoring manual [25] into a score ranging from 0 to 100, with a higher score indicating better QOL for functioning and global QOL. For cancer-specific symptoms, a higher score indicates more or worse symptoms and thus a poorer QOL.

Socio-demographic data collected in this study include age, ethnicity, education level, occupation, household income, marital status, number of children, breastfeeding history, and family history. Clinical data consisting of disease stage and comorbidity were extracted from the participants’ medical records. The patients were interviewed at five time points (first, third, fourth, fifth, and sixth visits) during the six-course therapy cycle. Data were collected personally via interview by the researcher. Time to complete the questionnaire was approximately 15–20 minutes.

### Analysis

Data were analyzed using the Statistical Package for Social Sciences (SPSS) version 18. Descriptive statistics were used to summarize the patients’ sociodemographic data, and differences between the characteristics of the intervention and control groups were evaluated using the chi square test. The effect of VCO consumption on QOL was determined by calculating the mean score difference between the intervention and control groups. An independent t-test was used to compare the QOL domains score between the intervention and control groups. P ≤ 0.01 was considered to be statistically significant.

## Results

### Characteristics of the study group

A total of 68 women who were diagnosed with stage III or IV breast cancer were asked to participate in this study. Sixty women (88.2%) completed the entire assessment process from the first cycle of chemotherapy to the sixth cycle. Five women (7.3%) withdrew from the intervention group because they were unable to take VCO, and three (4.5%) women from the control group refused to participate in the study because they were not feeling well. The final intervention group consisted of 30 women, and the control group consisted of 30 women. Characteristics of both the intervention and control groups are presented in Table 1. The age of the intervention group ranged from 31–73 years (mean 47.10 years), and those in the control group ranged in age from 30 to 64 years (mean 49.90 years). The majority of the patients were Malay (intervention group, 96.7%; control group, 80%) and had a secondary level of education (intervention group, 53.3%; control group, 36.7%). In the intervention group, 46.7% of participants were housewives, whereas in the control group 46.7% of participants were civil servants. Most participants had an income ranging between RM 1000 and 3000. Ninety percent of subjects in the intervention group were married compared to 83.3% in the control group. In both groups, 93.3% of patients breastfed their children. In the intervention and control groups, 40% and 20% of subjects, respectively, had a family history of cancer. Statistically, there were no significant differences between the intervention and control groups in terms of their demographic data (Table 1).

**Table 1:** **Sociodemographic data and clinical characteristics of the study sample (n = 60)**

Variables	Frequency (%)		P*
	Intervention group (n = 30)	Control group (n = 30)	
**Age group (years)**			0.386
< 40	6 (19.4)	9 (29.3)	
40–50	14 (48.3)	9 (29.3)	
> 50	10 (32.3)	12 (41.4)	
Mean (SD)	47.10 ( 8.2)	49.90 (11.5)	
Range	31–73	30–64	
**Race**			0.097
Malays	29 (96.7)	24 (80.0)	
Chinese	1 (3.3)	4 (13.3)	
Indian	–	2 ( 6.7)	
**Educational level**			0.131
None	–	3 (10.0)	
Primary	4 (13.3)	7 (23.3)	
Secondary	16 (53.3)	11 (36.7)	
College/University	10 (33.3)	9 (30.0)	
**Occupation**			0.694
Housewife	14 (46.7)	12 (40.0)	
Civil servant	13 (43.3)	14 (46.7)	
Private sector	3 (10.0)	4 (13.3)	
**Income**			0.209
< RM 1000	3 (10.0)	9 (30.0)	
RM 1000**-**RM3000	17 (56.7)	10 (33.3)	
**>**RM3000	10 (33.3)	11 (36.7)	
**Marital status**			0.385
Single	1 (3.3)	1 (3.3)	
Married	27 (90.0)	25 (83.3)	
Divorced	1 (3.3)	–	
Widowed	1 (3.3)	4 (13.3)	
**Number of children**			0.131
< 5	25 (83.2)	26 (86.7)	
> 5	5 (16.8)	4 (13.3)	
**Breast feeding**			0.945
No	2 (6.7)	2 (6.7)	
Yes	28 (93.3)	28 (93.3)	
**Family history of cancer**			0.128
No	18 (60.0)	24 (80.0)	
Yes	12 (40.0)	6 (20.0)	

*Pearson chi square test.

### Functioning and global QOL

Table 2 shows patients’ functioning and global QOL as measured by the EORTC QLQ-C30. All aspects of functioning showed improvement over time, and every functioning item had higher values in the intervention group compared to the control group at last chemotherapy cycle. Significant differences in mean functioning and global QOL scores between the intervention and control groups were also detected. At the sixth cycle of chemotherapy for global QOL has shown a significant different with a P value < 0.01 (*t-*stat: 3.325). It can be proven where the difference of the mean score for global quality of life at sixth chemotherapy cycle for the intervention group was 72.03 (SD: 12.81) while the control group was 60.34 (SD: 20.36). These results show more improvement in the intervention group than in the control group when comparing the mean score from the pre-assessment (first cycle) to the post-assessment (sixth cycle).

**Table 2:** Breast cancer patients’ functioning and global quality of life scores as measured by the EORTC QLQ-C30* (n = 60, 30 in each group)

Functioning	1st Chemotherapy cycle mean score (SD)		3rd Chemotherapy cycle mean score (SD)		4th Chemotherapy cycle mean score (SD)		5th Chemotherapy cycle mean score (SD)		6th Chemotherapy cycle mean score (SD)	
	Intervention	Control	Intervention	Control	Intervention	Control	Intervention	Control	Intervention	Control
**Physical**	69.69	73.57	68.72	68.72	70.53	75.18	78.12	74.48	83.00	75.18
	(18.42)	(27.11)	(16.39)	(18.81)	(16.23)	(15.00)	(12.40)	(19.28)	(12.17)	(15.93)
**Role**	58.05	67.35	60.77	59.19	60.76	67.25	69.21	64.96	77.98**	62.09**
	(25.06)	(35.99)	(22.19)	(30.41)	(21.35)	(20.16)	(17.54)	(19.07)	(19.42)	(22.67)
**Emotional**	60.77	60.77	62.93	64.36	66.09	66.12	71.23	65.81	76.09**	62.30**
	(20.35)	(29.78)	(14.87)	(26.72)	(15.53)	(21.12)	(15.52)	(19.59)	(14.86)	(20.92)
**Cognitive**	61.84	72.99	72.31	73.56	69.35	70.69	73.11	65.52	80.65**	64.37**
	(25.12)	(24.96)	(18.64)	(19.68)	(19.28)	(25.06)	(13.38)	(23.12)	(15.56)	(26.62)
**Social**	66.67	67.44	66.16	67.41	69.37	68.76	73.16	63.04	77.43	63.03
	(27.90)	(31.03)	(22.93)	(28.70)	(23.21)	(20.48)	(19.99)	(21.68)	(20.43)	(24.90)
**Global**	51.60**	65.79**	55.64**	71.54**	61.84	63.50	74.21	67.53	72.03**	60.34**
	(13.86)	(18.95)	(17.40)	(16.88)	(15.19)	(18.29)	(54.86)	(13.96)	(12.82)	(20.36)

*A higher value indicates a higher level of functioning and quality of life: min: 0, max: 100.

**Significant difference between intervention and control groups, P <0.01;

Independent *t-*test.

### Symptoms

For the symptoms measured using the EORTC QLQ-C30, there were no significant differences in mean symptom scores between the two groups at any cycle of the assessment (Table 3). However, by comparing mean score between the intervention group and the control group in the pre-assessment time point for all symptoms, the intervention group has indicates of a higher mean compared to the control group. Loss of appetite was the most detrimental symptom affecting the QOL for both groups. At the pre-assessment time point, the intervention group had a higher mean score for this symptom (50.53, SD: 33.20) compared to the control group (33.35, SD: 30.88). In addition, fatigue, dyspnea, and sleep difficulties were also lower at sixth cycle of chemotherapy as compared to the first cycle assessment in the intervention group. These results indicate that the QOL increased as the mean score of symptoms decreased.

**Table 3:** Breast cancer patients’ symptoms scores as measured by the EORTC QLQ-C30* (n = 60, 30 in each group)

Symptom	1st Chemotherapy cycle mean score (SD)		3rd Chemotherapy cycle mean score (SD)		4th Chemotherapy cycle mean score (SD)		5th Chemotherapy cycle mean score (SD)		6th Chemotherapy cycle mean score (SD)	
	Intervention	Control	Intervention	Control	Intervention	Control	Intervention	Control	Intervention	Control
**Fatigue**	49.34	36.38	44.26	39.93	46.06	40.56	45.44	41.71	45.88	47.26
	(22.75)	(24.50)	(11.69)	(22.94)	(13.77)	(15.76)	(15.29)	(15.59)	(16.10)	(17.58)
**Nausea and vomiting**	31.19	24.13	36.03	28.73	30.10	26.55	30.11	33.91	30.64	39.08
	(25.38)	(25.03)	(24.02)	(28.48)	(18.95)	(20.53)	(20.82)	(23.35)	(21.98)	(31.89)
**Pain**	34.03	28.16	34.97	29.87	37.09	29.30	34.40	64.35	34.94	41.95
	(23.34)	(26.39)	(16.86)	(21.07)	(17.05)	(21.66)	(18.72)	(148.92)	(20.35)	(20.72)
**Dyspnoea**	22.57	14.94	21.51	12.63	11.49	15.58	18.82	19.53	18.29	20.68
	(26.37)	(23.30)	(25.17)	(18.71)	(18.72)	(18.41)	(23.88)	(22.74)	(25.61)	(24.26)
**Sleep difficulties**	36.55	24.12	38.73	26.42	37.09	33.32	33.86	35.61	31.71	39.06
	(26.33)	(30.73)	(24.50)	(27.29)	(22.67)	(28.18)	(22.16)	(23.47)	(25.23)	(21.96)
**Loss of appetite**	50.53	33.35	49.47	44.25	48.91	43.68	41.38	43.66	40.85	44.82
	(33.20)	(30.88)	(27.05)	(31.60)	(19.72)	(25.38)	(23.14)	(23.76)	(23.52)	(32.47)
**Constipation**	31.16	28.75	37.64	25.28	38.16	24.14	38.70	34.47	40.86	41.38
	(27.12)	(35.34)	(23.38)	(27.69)	(19.83)	(28.05)	(24.50)	(30.20)	(26.83)	(31.71)
**Diarrhoea**	12.90	10.34	17.22	11.49	17.19	16.09	19.34	17.23	26.86	20.69
	(20.50)	(20.13)	(22.57)	(20.46)	(20.84)	(22.92)	(22.39)	(22.92)	(59.83)	(27.34)
**Financial difficulties**	32.25	26.43	25.81	28.73	33.33	27.58	36.56	31.02	37.62	34.48
	(31.61)	(30.06)	(25.40)	(31.78)	(31.04)	(30.95)	(29.01)	(29.46)	(29.50)	(31.48)

*A higher value indicates a higher level of functioning and quality of life: min: 0, max: 100.

**Significant different significant difference between intervention and control groups, P < 0.01;

Independent *t-*test.

### Breast-specific scores

The QOL scores for breast cancer patients measured by the EORTC QLQ-BR23 are shown in Table 4. Except for sexual enjoyment, improvements were seen in all other functioning scores (i.e., body image, sexual function and future perspective) compared to the first cycle of chemotherapy in the intervention group. The mean score for sexual function improved in the intervention group between the first and the sixth cycle, but unfortunately, the mean differences were not significant between groups (P > 0.01; *t-*stat: -1.507). At the fifth chemotherapy cycle, the intervention group had a higher mean score for sexual function compared to the control group (53.83, SD: 27.58 vs. 38.54, SD: 34.04). For symptom status, none of the symptoms (P > 0.01) has shown significant mean score differences between groups at any cycles of the chemotherapy. By comparing between the mean score for intervention group of each symptoms, the symptom of breast (15.59, SD: 17.58 vs 15.08, SD: 12.61) and systematic therapy side effects (38.80, SD: 19.99 vs 38.24, SD: 18.83) has indicate lower mean score at the sixth cycle prior to the first cycle. The side effects of systemic therapy in the control group had the highest mean score of symptoms, which could diminish the QOL among breast cancer patients. Although the score for upset by hair loss was somewhat higher in the intervention group than the control group after six cycles of chemotherapy, the difference between the groups was not statistically significant.

**Table 4:** **Breast cancer patients’ functioning and symptoms scores as measured by the EORTC QLQBR23 (n = 60, 30 in each group)**

	1st Chemotherapy cycle mean score (SD)		3rd Chemotherapy cycle mean score (SD)		4th Chemotherapy cycle mean score (SD)		5th Chemotherapy cycle mean score (SD)		6th Chemotherapy cycle mean score (SD)	
	Intervention	Control	Intervention	Control	Intervention	Control	Intervention	Control	Intervention	Control
Functioning*										
**Body image**	64.52	73.93	67.75	68.70	67.18	75.02	72.66	71.75	75.59	68.72
	(21.71)	(23.25)	(18.24)	(24.24)	(19.87)	(17.71)	(21.16)	(18.23)	(24.81)	(18.91)
**Sexual functioning**	46.82	33.83	47.37	32.16	54.90	39.09	53.83	38.54	53.85	42.55
	(31.23)	(35.49)	(30.26)	(28.93)	(28.97)	(31.96)	(27.58)	(34.04)	(28.85)	(34.17)
**Sexual enjoyment**	34.43	27.59	25.85	19.55	19.33	24.14	22.60	25.28	20.44	27.59
	(32.86)	(37.91)	(29.58)	(30.31)	(25.55)	(30.81)	(29.14)	(30.49)	(28.21)	(31.04)
**Future perspective**	31.08	48.28	37.53	48.31	37.58	48.35	50.60	50.69	53.90	56.48
	(24.30)	(36.37)	(25.54)	(35.27)	(22.49)	(29.14)	(25.80)	(29.18)	(22.42)	(25.51)
**Symptom+**										
**Arm**	24.72	17.99	25.43	19.53	27.23	24.51	27.94	22.97	27.94	24.88
	(22.72)	(18.39)	(17.73)	(21.13)	(20.86)	(22.29)	(17.42)	(16.51)	(18.35)	(17.97)
**Breast**	15.59	12.55	10.74	14.88	10.21	18.10	13.71	16.37	15.04	20.68
	(17.58)	(18.71)	(11.82)	(19.08)	(8.26)	(18.38)	(9.76)	(12.09)	(12.61)	(16.75)
**Systematic therapy side effect**	38.80	33.18	39.02	37.29	39.79	39.91	42.10	41.20	38.24	48.65
	(19.99)	(16.77)	(14.73)	(16.87)	(13.99)	(19.25)	(17.99)	(19.68)	(18.83)	(18.23)
**Upset by hair loss**	25.28	31.03	24.51	16.67	34.48	25.28	34.84	28.96	36.77	36.77
	(30.13)	(36.42)	(24.73)	(27.46)	(31.48)	(31.70)	(24.45)	(30.31)	(28.82)	(31.31)

*Scores range from 0 to 100, with higher scores representing a higher level of functioning.

+Scores range from 0 to 100, with higher scores representing a higher level of symptoms.

**Significant different, P <0.01;

## Discussion

This study was designed to evaluate the effectiveness of VCO on the QOL of patients with breast cancer throughout six cycles of chemotherapy. The results showed that the functional status and global QOL were improved in the intervention group, with significant mean score differences between groups. Additionally, symptoms of fatigue, dyspnea, sleep difficulties and loss of appetite were also decreased in the intervention group, measured at the sixth cycle of chemotherapy. For breast-specific scores, there was improvement in all other patients’ functioning scores likes body image, sexual function and future perspective. Breast symptoms and side effects of systemic therapy showed significant mean score differences between groups at the sixth cycle of chemotherapy.

The findings of this study showed that women had a satisfactory score on functioning and global quality of life after consumption of VCO. In fact, breast cancer and its treatment, especially chemotherapy, can disrupt the QOL of breast cancer patients [12, 13, 26]. In addition, a study done by Vardy [15] & Lemieux *et al*., [27] reported that quality of life was most affected after patients experienced side effects from breast cancer treatments. At the sixth chemotherapy cycle, mean score for global QOL for the intervention group was 72.03 (SD: 12.82) while the control group was only 60.34 (SD: 20.36). This finding is contrary to the study performed by Montazeri *et al*., [28], whereby the global health score was reduced. Nevertheless, the results are similar to Costanzo et al., [29], which showed improvement in physical functioning. It is believed that consumption of VCO helped in increasing the energy level as well as maintaining physical function among the breast cancer patients. The effects of VCO in terms of energy, fats and protein metabolism have been demonstrated in the study carried out by Soerjodibroto [22]. The study highlighted that VCO had a positive effect on immune responses among HIV positive patients which showed improvements and maintained stable throughout the study. Lauric acid which is a disease- fighting substance that has many health benefits [30–32], is abundant in coconut oil. Fife [33] also mentioned that coconut oil improves nutrient absorption, boosts energy, possesses potent antimicrobial properties, and improves energy metabolism in the brain. There have been emerging fundamental evidences to explain the positive clinical effects of VCO on human health.

For the symptom scores evaluated in this study, no significant differences between the two groups were detected. Thus, the improvement of the symptoms scores were presented such as fatigue, dyspnea, sleep difficulties and loss of appetite at the sixth chemotherapy cycle. Serious side effects of chemotherapy often are perceived by patients as distress factors that contribute to poor QOL [12, 26]. For example, the most commonly reported symptom that affects QOL was loss of appetite. During the pre-assessment, the intervention group had a higher mean score for this symptom (50.53, SD: 33.20) than the control group (33.35, SD: 30.88). However, after VCO supplementation, the mean score of the intervention group declined and no significant difference between the two groups was detected. Coconut oil is unique, known as medium-chain triglycerides that are usually used to improve patients’ nutritional status [32, 33]. In addition, maintaining a good appetite can promote wound healing and encourage a speedy recovery from illness [7].

The QOL scores of breast cancer patients as measured by the EORTC QLQ-BR23 in this study showed improvement in several measures of function such as body image, future perspective and sexual function in the intervention group. Compared to a study performed by Montazeri *et al*., [34], instead use found that most of the function mean scores decreased especially sexual function as a result of premature menopause following adjuvant systemic therapy in breast cancer patients. Physical and emotional status have been shown to affect sexuality, including negative body image, feelings of sexual unattractiveness, loss of femininity, depression, and anxiety following breast cancer diagnosis [35]. Ganz *et al.,* [36] found that sexual functioning was worse for women who received chemotherapy. A similar result has been noted in another study from University of Sto. Tomas unveiled the beneficial effects of VCO on health and sex life. 13% of the VCO takers (189 participants) experienced becoming sexually active in the whole duration of their participation in the VCO study [37].

The current study was limited by the small number of breast cancer patients in Kelantan, Malaysia. Therefore, a large, multi-state comparison with varying demographics should be conducted in Malaysia. Furthermore, additional VCO intervention studies should be performed to confirm the specific beneficial effects of VCO that were detected in this study.

## Conclusion

This study revealed improved functional and global QOL of breast cancer patients who consumed VCO during the six cycle chemotherapy treatment and showed significant differences in functional and global QOL between the intervention and control groups. VCO may help in promoting the functional and global QOL of breast cancer patients who are undergoing chemotherapy. These results also suggest that VCO consumption could reduce the women’s symptoms and improve several items of functional status such as body image, future perspective and sexual function. Based on the results, there are certainly good prospect of utilizing VCO as supplementation among breast cancer patients towards achieving a positive QOL.
